# Development of a “Signal-On” Fluorescent Aptasensor for Highly Selective and Sensitive Detection of ZEN in Cereal Products Using Nitrogen-Doped Carbon Dots Based on the Inner Filter Effect

**DOI:** 10.3390/bios14070347

**Published:** 2024-07-17

**Authors:** Qi Sun, Yuting Zhou, Miaomiao Ma, Fuyan Zhang, Shuang Li, Zhuoer Chen, Yu Fang, Tao Le, Fuguo Xing

**Affiliations:** 1 Chongqing Key Laboratory of Conservation and Utilization of Freshwater Fishes, College of Life Sciences, Chongqing Normal University, No. 37 Chengzhong Road, Shapingba District, Chongqing 401331, China; 2022210513077@stu.cqnu.edu.cn (Y.Z.); 2023100513032@stu.cqnu.edu.cn (M.M.); 2022210513071@stu.cqnu.edu.cn (F.Z.); 2022210513061@stu.cqnu.edu.cn (S.L.); 2022110513026@stu.cqnu.edu.cn (Z.C.); 2022210513053@stu.cqnu.edu.cn (Y.F.); letao@cqnu.edu.cn (T.L.); 2 Key Laboratory of Agro-Products Quality and Safety Control in Storage and Transport Process, Ministry of Agriculture and Rural Affairs, Beijing 100193, China; 3 Institute of Food Science and Technology, Chinese Academy of Agricultural Sciences, Beijing 100193, China

**Keywords:** zearalenone, nitrogen-doped carbon dots, fluorescent aptasensor, detection mechanism, cereal products

## Abstract

This study aimed to develop a novel fluorescent aptasensor for the quantitative detection of zearalenone (ZEN), addressing the limitations of conventional detection techniques in terms of speed, sensitivity, and ease of use. Nitrogen-doped carbon dots (N-CDs) were synthesized via the hydrothermal method, resulting in spherical particles with a diameter of 3.25 nm. These N-CDs demonstrated high water solubility and emitted a bright blue light at 440 nm when excited at 355 nm. The fluorescence of N-CDs was quenched by dispersed gold nanoparticles (AuNPs) through the inner filter effect, while aggregated AuNPs induced by NaCl did not affect the fluorescence of N-CDs. The aptamer could protect AuNPs from NaCl-induced aggregation, but the presence of ZEN weakened this protective effect. Based on this principle, optimal conditions for ZEN detection included 57 mM NaCl, 12.5 nM aptamer concentration, incubation of AuNPs with NaCl for 15 min in Tris-EDTA(TE) buffer, and incubation of aptamer with ZEN and NaCl for 30 min. Under these optimized conditions, the “signal-on” fluorescent aptasensor for ZEN detection showed a linear range of 0.25 to 200 ng/mL with a low detection limit of 0.0875 ng/mL. Furthermore, the developed aptasensor exhibited excellent specificity and could rapidly detect ZEN in corn flour samples or corn oil, achieving satisfactory recovery rates ranging from 84.7% to 108.6%. Therefore, this study presents an economical, convenient, sensitive, and rapid method for accurately quantifying ZEN in cereal products.

## 1. Introduction

Mycotoxin contamination represents a significant global food safety concern [1]. Zearalenone (ZEN), an estrogenic mycotoxin produced by Fusarium species, ranks among the most prevalent mycotoxins and poses substantial risks to food and feed safety [2]. Its accumulation in the human body through the food chain presents a considerable health hazard. Due to its potent toxicity and persistent metabolism, the European Commission has established a tolerable daily intake for ZEN at 0.25 μg/kg of body weight [3]. Therefore, the development of straightforward, cost-effective methods for the rapid detection and sensitive monitoring of ZEN is crucial for ensuring food safety and protecting human health. Currently, primary methods for detecting ZEN in China include instrumental detection and immunoassays. Instrumental methods such as liquid chromatography-mass spectrometry (LC-MS/MS) [4,5] and matrix-assisted laser desorption/ionization time-of-flight mass spectrometry (MALDI-TOFMS) [6] offer high sensitivity and accuracy but suffer from high cost, complexity, and long detection duration. Immunoassays, while providing speed and convenience, often utilize antibodies as recognition elements, rendering them expensive, time-consuming, and prone to false positives [7].

Aptamers, also known as aptabodies or chemical antibodies, serve as robust chemical competitors to antibodies [8]. These structured nucleic acids are obtained through in vitro screening methods such as the systematic evolution of ligands by exponential enrichment (SELEX) [7,9]. Aptamers are characterized by high specificity and selectivity in binding to target molecules, offering advantages such as easy modification, rapid synthesis, and cost-effectiveness, which are often lacking in antibodies [10]. Aptamers readily adopt stable spatial structures like helices, stem-loops, and clovers, facilitating tight binding to targets via van der Waals forces and hydrogen bonds [11]. These structures can differentiate similar substances, making aptamers valuable recognition elements in biosensor applications [12]. Gold nanoparticles (AuNPs) are widely used in the construction of optical biosensors due to their ease of preparation, excellent biocompatibility, chemical stability, and unique optical properties [13]. Consequently, various AuNPs-based optical sensors have been extensively developed and applied for detecting contaminants in environmental and biological fields [14,15]. Fluorescent sensors, in particular, offer distinct advantages such as high sensitivity, ease of operation, and on-site rapid monitoring compared to other optical sensing methods [16]. The detection of pollutants using fluorescent aptasensors is a prominent focus in current literature, with numerous studies reporting satisfactory results [17]. Our research group has contributed significantly to this field by developing a variety of fluorescent aptasensors for mycotoxin detection [18,19]. However, a practical challenge in the applications of certain fluorescent sensors lies in the synthesis of diverse nanomaterials. The synthesis of signal elements required for fluorescent sensors using simple or common methods has become a critical yet often overlooked issue during sensor construction and practical detection.

The critical role of fluorescent probes in achieving sensitive detection of targets for fluorescent sensors is well recognized. Currently, carbon quantum dots have emerged as leading candidates for fluorescence detection compared to other fluorescent probes [20]. This is primarily due to their exceptional photoluminescence, photostability, strong biocompatibility, affordability, and straightforward preparation and functionalization methods [21]. Therefore, this study aimed to synthesize nitrogen-doped carbon quantum dots (N-CDs) through a one-step solvothermal method to serve as fluorescent donors. AuNPs, prepared using the standard citrate reduction method, were employed as fluorescent receptors, with aptamers integrated as specific recognition elements for mycotoxins. The resulting fluorescent aptasensor holds promise as a straightforward and rapid for detecting and quantifying ZEN in real samples.

## 2. Materials and Methods

### 2.1. Chemical Reagents

All chemical reagents were of analytical grade and purchased from Shanghai Aladdin Biochemical Technology Co., Ltd. (Shanghai, China), without prior purification, unless otherwise specified. Mycotoxin standards, including ochratoxin A (OTA), aflatoxin B_1_ (AFB_1_), deoxynivalenol (DON), patulin (PAT), and ZEN, were obtained from Shanghai Yuanye Biotechnology Co., Ltd. (Shanghai, China). The ZEN aptamer sequence (5′-TCATCTATCTATGGTACATTACTATCTGTAATGTGATATATG-3′) was sourced from the literature [22] and synthesized by Sangon Biotech Co., Ltd. (Shanghai, China). Ultrahigh purity water (18.2 MΩ cm^−1^, Milli-Q, Darmstadt, Germany) was used for the preparation of all solutions.

### 2.2. Material Characterization

The surface morphology and microstructural properties of the prepared N-CDs were examined using a transmission electron microscope (TEM, JEM-2100F, JEOL, Tokyo, Japan). The fluorescence lifetime of N-CDs was measured with a photoluminescence spectrometer (FLS1000, Edinburgh Instruments, Edinburgh, England) to determine the average fluorescence lifetime and decay model of fluorescence intensity. Fourier transform infrared (FTIR) spectra of N-CDs were obtained using a Nicolet-iS10 FTIR instrument (Thermo Scientific, Waltham, MA, USA) equipped with an attenuated total reflectance accessory. The spectra were recorded over a range of 500–4000 cm^−1^ with 16 scans and a resolution of 8 (0.964 cm^−1^). The chemical compositions on the material surface were analyzed by X-ray photoelectron spectroscopy (XPS, Thermo Fisher Scientific, Model ESCALAB 250 XI XPS, Waltham, MA, USA). Fluorescence and ultraviolet-visible (UV-Vis) spectra of N-CDs were collected using an F-7100 fluorescence spectrophotometer (Hitachi, Tokyo, Japan) and a Hitachi UV-Vis UH-5300 spectrophotometer, respectively.

### 2.3. Preparation of AuNPs

The AuNPs were prepared following the trisodium citrate reduction method as previously reported [23]. In brief, 2 mL of 1% trisodium citrate dihydrate was quickly added to 100 mL of 0.01% chloroauric acid solution, and the mixture was boiled for 5 min until a wine-red color developed. Subsequently, the mixture was allowed to cool to room temperature before being centrifugated at 13,000 rpm for 15 min to remove excess citrate. Finally, the resulting sediment was then resuspended in distilled water and stored at 4 °C.

### 2.4. Synthesis of N-CDs

**Scheme 1:**
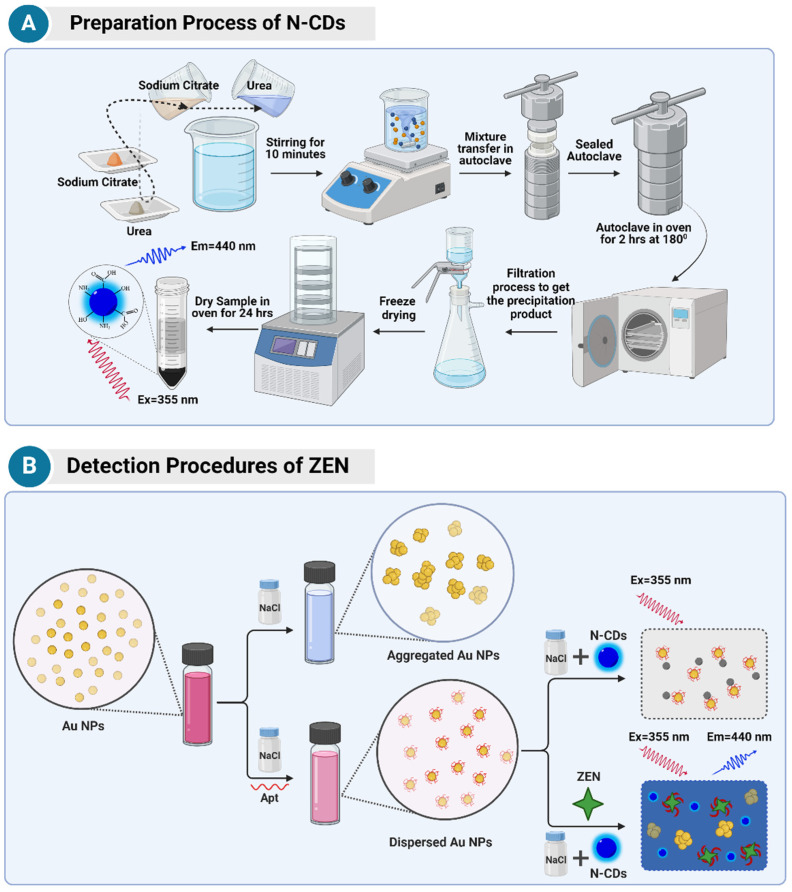
(**A**) Flowchart depicting the hydrothermal synthesis process of nitrogen-doped carbon dots (N-CDs). (**B**) Schematic diagram of the assay of zearalenone (ZEN).

The synthesis of N-CDs has been described in the literature with a reference [24], and the specific procedure is illustrated in Scheme 1A. In short, 1.970 g of trisodium citrate and 2.413 g of urea were finely ground into a white powder, dissolved in 20 mL of water, and then stirred for 10 min. The solution was then transferred to a 50 mL Teflon-lined autoclave for a solvent-heated reaction at 180 °C for 2 h. Following the filtration process, the precipitation product was freeze-dried for 24 h to obtain N-CDs, which were then dissolved in a buffer solution to the required concentration before use.

### 2.5. Fluorescent Aptasensing of ZEN

The trials involving the fluorescent aptasensing of ZEN were conducted in a fixed final volume of 300 µL. Initially, a specific concentration of ZEN was mixed with the optimal level of N-CDs and incubated at 25 °C for an appropriate duration. Subsequently, 140 µL of AuNPs were added to the mixture and incubated at 25 °C for 30 min. Next, 10 µL of the NaCl solution (57 mM) and 60 µL of the N-CDs solution (0.4 mg/mL) were added to the mixture and incubated for 15 min at 25 °C. Finally, 300 µL of the resulting samples were transferred to a 0.75 mL quartz cell, and fluorescence measurements were conducted. Changes in fluorescence intensity were calculated using the formula ∆F = F − F_0_, where F and F_0_ represented the fluorescence intensities of the sensor in the presence or absence of ZEN, respectively.

### 2.6. Specificity Test

The specificity of the developed fluorescent aptasensor was assessed by testing its response to several potential mycotoxins commonly found in cereal products, including OTA, AFB_1_, DON, and PAT. Different concentrations of each interfering mycotoxin were separately mixed with the sensing system, and the resulting fluorescence intensities were measured and recorded.

### 2.7. Detection of Real Samples

The feasibility of the fabricated fluorescent aptasensor based on N-CDs and AuNPs was evaluated using both corn flour and corn oil. In the spiked recovery trial, a known quantity of ZEN was added to the aforementioned corn products, followed by fluorescent detection as described above. High-performance liquid chromatography (HPLC) analysis served as the standard method for quantifying ZEN concentrations in real samples.

### 2.8. Statistical Analysis

Statistical analyses were conducted using the SPSS version 22.0 (SPSS Inc., Chicago, IL, USA). All experiments were performed at least three times. Data were presented as means ± standard deviations (SDs). Statistical significances were conducted using one-way analysis of variance (ANOVA), followed by Tukey’s multiple comparison test.

## 3. Results and Discussion

### 3.1. Morphology and Micro-Structure Characterization of N-CDs

**Figure 1:**
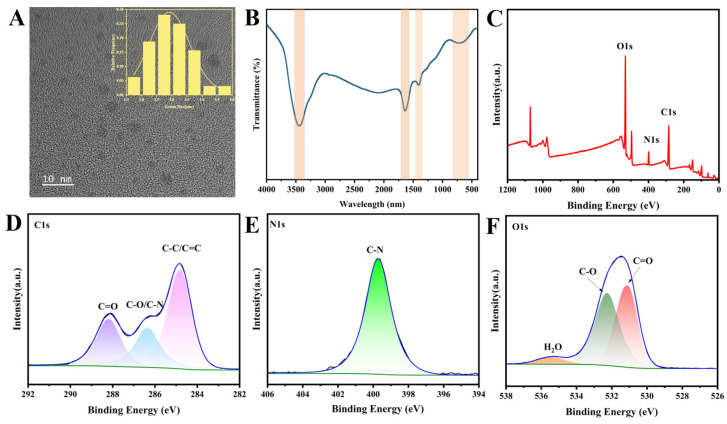
(**A**) Transmission electron microscopy image of N-CDs, with inset showing the particle size distribution; (**B**) Fourier-transform infrared spectroscopy spectrum of N-CDs. (**C**) X-ray photoelectron spectroscopy full survey spectrum of N-CDs. (**D**) High-resolution spectra of C1s, (**E**) High-resolution spectra of N1s, and (**F**) High-resolution spectra of O1s. N-CDs: nitrogen-doped carbon dots.

The TEM image (Figure 1A) of N-CDs showed the as-synthesized blue CDs with good mono-dispersity and quasi-spherical morphology. The size distribution histograms (inset of Figure 1A) confirm that the N-CDs range in size from 2.35 to 4.50 nm, with an average diameter of 3.25 nm. Elemental analysis indicated that the nitrogen content of the CDs reached 7.82%, suggesting the successful preparation of nitrogen-doped CDs. The functional groups and chemical composition were analyzed using FTIR and XPS. The FTIR spectrum (Figure 1B) displayed a broad peak at approximately 3440 cm^−1^, indicating the stretching vibrations of the O-H and N-H bonds. Additionally, a prominent peak observed at 1643 cm^−1^ corresponded to C=O stretching vibration, while a signal at 1406 cm^−1^ was assigned to a C-NH vibration [25]. The broad band in the range of 550~850 cm^−1^ was characteristic of the C-O stretching vibration, indicating the presence of functional groups that can enhance the aqueous solution stability and improve the hydrophilicity of N-CDs [25]. The XPS full survey spectrum (Figure 1C) of the prepared N-CDs exhibited three peaks at 292 eV (C1s), 403 eV (O1s), and 537 eV (N1s) [26]. The C1s spectrum (Figure 1D) revealed four chemical states of carbon: C-C/C=C at 284.9 eV, sp3 C from C-O at 285.2 eV, C-N at 286.4 eV, and C=O at 288.2 eV, respectively. The N 1s spectrum (Figure 1E) displayed a peak at 400.2 eV corresponding to the C-N bond. In addition, the peaks at 530.9 eV, 532.3 eV, and 535.2 eV in the O 1s spectrum (Figure 1F) could be attributed to C=O, C-O, and H_2_O [27,28], respectively.

### 3.2. Optical Properties of As-Synthesized N-CDs

**Figure 2:**
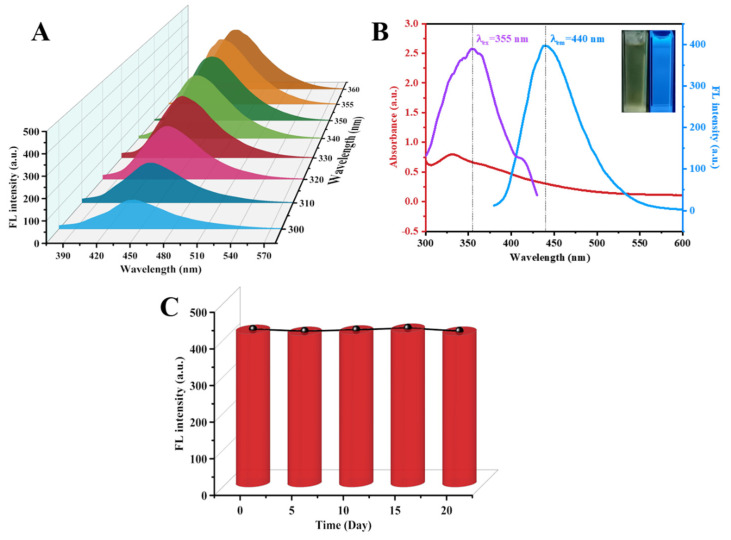
(**A**) Excitation-emission spectra of fluorescence for N-CDs. (**B**) UV-Vis absorption and fluorescence spectra of N-CDs. The inset displays N-CDs in aqueous solution under sunlight (left) and 365 nm UV light (right) irradiation. (**C**) Fluorescence stability test of N-CDs. N-CDs: nitrogen-doped carbon dots.

The optical properties of the obtained N-CDs were extensively analyzed using fluorescence spectroscopy and UV-Vis spectroscopy. The UV-Vis absorption spectrum of N-CDs exhibited a prominent peak at 330 nm (Figure 2B), attributed to the n-π* transition of the C=O functional group in N-CDs [29]. Despite variations in fluorescence intensity, the fluorescence emission peak position of N-CDs remained consistent across different excitation wavelengths (Figure 2A). The optimal excitation and emission wavelengths for the synthesized N-CDs were determined to be 355 nm and 440 nm, respectively (Figure 2B). Consequently, the aqueous solution of N-CDs appeared as a brownish-yellow color under sunlight, but transitioned to blue when exposed to 365 nm UV light (inset in Figure 2B). Additionally, the obtained N-CDs demonstrated remarkable radiation resistance, with only a slight decrease in fluorescence intensity observed after 20 days of exposure to sunlight (Figure 2C). This resilience can be attributed to the stable chemical structure and surface functional groups present in N-CDs, including hydroxyl, carbonyl, and amino groups [30]. These findings underscored the excellent photo-stability of the prepared N-CDs, making them highly promising candidates for future applications in fluorescent detection.

### 3.3. Fabrication and Detection Principle of Aptasensor

The AuNPs used were surface stabilized with citrate, possessing a negative charge. This charge ensured that the AuNPs remained stable and did not aggregate. Consequently, they exhibited a uniform dispersion in solution, which was characterized by a wine-red color [31]. Upon addition of NaCl to the AuNPs solution, the negative charge on the surface of AuNPs surface was neutralized by Na^+^, leading to non-crosslinked aggregation and a color change from wine-red to blue-violet in the mixed solution (Scheme 1B). Aptamers, as single-stranded DNA, could be adsorbed onto the surface of AuNPs through van der Waals as well as hydrophobic interactions [32], thereby providing protection from aggregation at high NaCl concentrations. Upon the introduction of N-CDs, their inherent fluorescence was efficiently quenched by the dispersed AuNPs. This interaction led to a decrease in fluorescence intensity, indicating the strong interaction between the N-CDs and AuNPs [33]. When ZEN was introduced into the system, the aptamer, known for its high affinity and anti-interference properties, was selectively bound to the ZEN molecule with high specificity, prompting the ZEN to detach from the surface of the AuNPs. This detachment enabled the AuNPs to aggregate in high NaCl concentrations. Consequently, the fluorescence of N-CDs was not quenched by the aggregated AuNPs, and the fluorescence in the reaction system was gradually restored and enhanced with increasing ZEN concentration.

**Figure 3:**
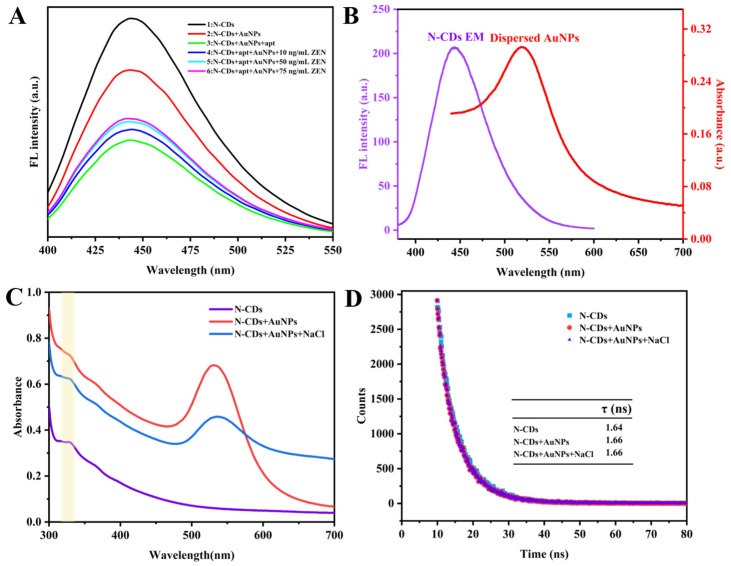
(**A**) Fluorescence emission spectra showing (1) N-CDs, (2) N-CDs + AuNPs, (3) N-CDs + AuNPs + Apt, and (4–6) N-CDs + AuNPs + Apt with different concentrations of ZEN. (**B**) Fluorescence emission spectrum of N-CDs and absorption spectra of dispersed AuNPs. (**C**) Absorption spectra. (**D**) Fluorescence lifetime plots of N-CDs, N-CDs + AuNPs and N-CDs + AuNPs + NaCl. N-CDs, nitrogen-doped carbon dots; AuNPs, gold nanoparticles; Apt, aptamer.

To verify the feasibility of the proposed theory, fluorescence spectroscopy analysis of different reaction systems was conducted. In the presence of a high-concentration salt solution (specifically, 57 mM NaCl), the solution of N-CDs exhibited a clear fluorescence peak at a wavelength of 440 nm, as indicated by curve 1 in Figure 3A. Upon the subsequent addition of AuNPs, there was a significant decrease in the fluorescence intensity of the N-CDs (curve 2 in Figure 3A), attributed to the fluorescence quenching property of AuNPs. Upon coating with aptamers, AuNPs acquired a higher density of negative charges through interaction with the nitrogen atoms of the aptamer bases [34]. This enhanced the dispersion of AuNPs in high-concentration NaCl solutions, further impacting the fluorescence quenching of N-CDs (curve 3 in Figure 3A). Upon addition of ZEN, aptamers, known for their high affinity, are selectively bound to ZEN molecules, leading to the aggregation of certain AuNPs within high-concentration salt solutions. This aggregation occurred in the absence of aptamer protection, which in turn weakened the fluorescence quenching effect of AuNPs on N-CDs. Consequently, the fluorescence intensity in the reaction system gradually increased with higher concentrations of ZEN (curves 4–6 in Figure 3A). Interestingly, neither the aptamer sequence nor the ZEN molecule alone exerted a significant impact on the fluorescence intensity of N-CDs. The behavior of fluorescence quenching and recovery in the developed aptasensor primarily depended on the status of AuNPs. The aggregation or dispersion of these AuNPs was significantly influenced by the concentration of ZEN. Thus, the proposed aptasensor demonstrated its capability to detect ZEN through fluorescence analysis. Additionally, the UV-Vis absorption spectrum of AuNPs showed a significant overlap with the fluorescence emission spectrum of N-CDs (Figure 3B). Additionally, the UV-visible absorption peak of N-CDs (Figure 3C) and their fluorescence lifetime (Figure 3D) remained unaffected by the presence and the varying states of AuNPs. These results suggest that the emitted fluorescence of N-CDs might be absorbed by AuNPs, leading to a quenching process that can be rationalized by the inner filter effect.

### 3.4. Optimization of the Detection Conditions

**Figure 4:**
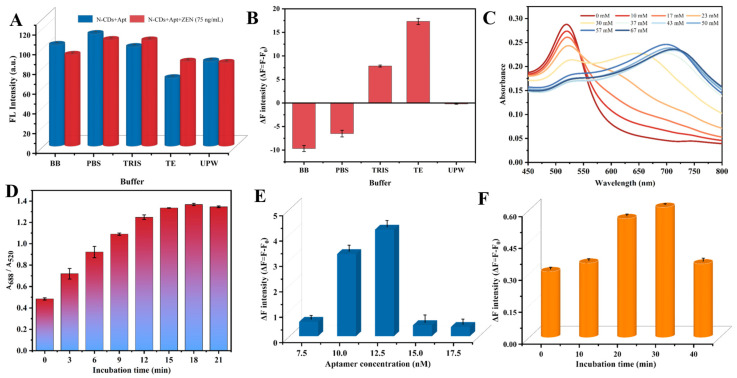
(**A**) Fluorescence intensity of the aptasensor with different buffer solutions in the presence and absence of ZEN. (**B**) Changes in fluorescence intensity during aptasensor optimizations; (**C**) concentrations of NaCl; (**D**) incubation time of AuNPs and NaCl; (**E**) concentrations of aptamer; (**F**) incubation time of aptamer and ZEN. AuNPs, gold nanoparticles; ZEN, Zearalenone.

The aptasensing conditions were optimized to achieve optimal assay performance. The selection of buffer is critical for aptasensing assays, as its composition can significantly influence binding properties in different environments. The highest fluorescence quenching-recovery values were achieved when Tris-EDTA (TE) buffer was selected as the optimal buffer (Figure 4A,B). Furthermore, the concentration of NaCl plays a crucial role in affecting the sensitivity of the aptasensor. As NaCl concentration gradually increased, the color of AuNPs shifted from wine-red to blue. Simultaneously, the absorbance gradually decreased at 520 nm and increased at 688 nm, reaching saturation at a NaCl concentration of 57 mM (Figure 4C). Beyond a reaction time of 15 min between AuNPs and NaCl, the ratio of absorbance values (A_688_/A_520_) tends to stabilize (Figure 4D), indicating that 15 min might be sufficient for complete aggregation of AuNPs under the influence of NaCl. Additionally, the optimization revealed that the most significant increase in fluorescence signal within the aptasensing system was observed at an aptamer concentration of 12.5 nM (Figure 4E) and an incubation time of 30 min between the aptamer and ZEN (Figure 4F).

### 3.5. Aptasensing Performance and Applications

**Figure 5:**
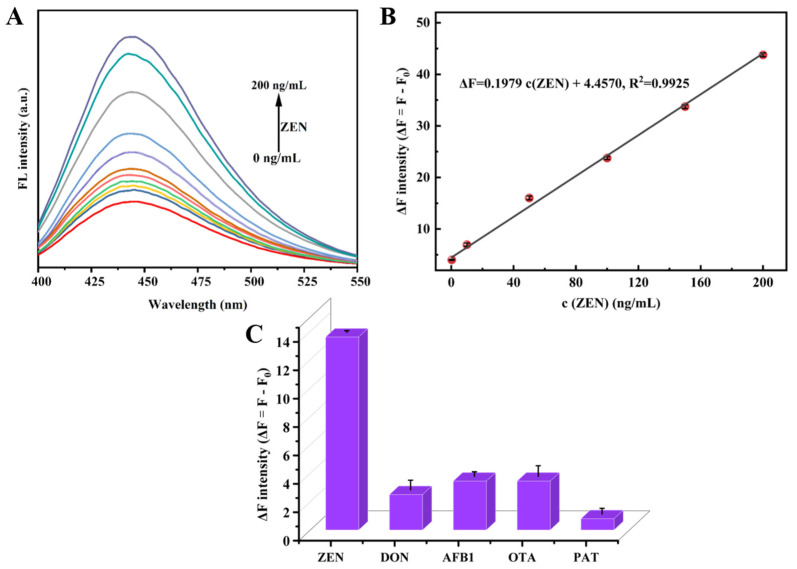
(**A**) Fluorescence spectra depicting the detection of different concentrations of ZEN (0–200 ng/mL). (**B**) Calibration curve correlating fluorescence intensity changes (ΔF = F − F_0_) under optimal aptasensing conditions. (**C**) Selectivity evaluation of the fabricated aptasensor. ZEN, zearalenone.

The performance of the fabricated aptasensor was further evaluated under the optimized detection conditions. Figure 5A shows the fluorescence emission spectra after different concentrations of ZEN were introduced into the aptasensing system and incubated for 30 min, the fluorescence signal increased gradually with rising ZEN concentration. The change in fluorescence intensity (ΔF = F − F_0_) was found to be directly proportional to the ZEN concentration within the range of 0.25–200 ng/mL. The linear equation derived from the data was ΔF = 0.1979c + 4.4570 with a high regression coefficient (R^2^ = 0.9925) (Figure 5B). The limit of detection (LOD) of the prepared aptasensor was calculated to be as low as 0.0875 ng/mL based on the triple standard deviation of 10 blank measurements. 

When compared with other methods for ZEN detection (Table 1), the aptasensor developed in this study demonstrated several advantages including a broad detection linear range and high sensitivity. As described, the prepared N-CDs exhibited several remarkable fluorescent characteristics, including high quantum yield, and excellent photostability. These properties arise from the quantum confinement effect and the presence of surface functional groups. In the case of nitrogen-doped carbon dots, this results in bright and tunable fluorescence. Moreover, this method offered the benefits of simple operation and low detection costs. The proposed sensor demonstrated a high level of specificity and minimal interference from potential contaminants such as DON, AFB_1_, OTA, and PAT (Figure 5C), largely due to the strong binding affinity between the aptamer and its intended target. Under the consistent ZEN concentration of 50 ng/mL, there was no notable variation in the ΔF value among the eight distinct experimental groups, with a relative standard deviation (RSD) of less than 2.8%. These results suggest that the N-CDs-based fluorescent sensor shows reliable reproducibility. The fluorescence sensor, utilizing the stable fluorescence of the prepared N-CDs, demonstrated consistent results over a 30-day testing period. The relative standard deviations of the fluorescence values for both systems, N-CDs + Apt and N-CDs + Apt + ZEN, were found to be less than 6%. For intra-assay recovery, the recoveries ranged from 96.9% to 105.8% with the highest coefficient of variation (CV) at 6.69% (Table 2). These results were consistent with those obtained using the conventional HPLC method (Table 2), where the correlation coefficient between the two assays was notably high at 0.9951. Therefore, our developed fluorescence aptasensor utilizing the internal filtering effect of N-CDs holds significant potential for the detection of ZEN in real samples.

## 4. Conclusions

A novel, sensitive, economical, and simple fluorescent aptasensor was successfully developed for the rapid detection of ZEN in real samples. N-CDs were used as the fluorescent donors, while AuNPs functioned as the fluorescent acceptors in this study, enabling fluorescence quenching through the inner filter effect. The synthesized N-CDs exhibited a spherical structure with good water solubility and demonstrated excitation-independent fluorescence behavior, emitting bright blue fluorescence with excellent stability. The presence of NaCl or aptamer changed the state of AuNPs, thereby influencing their ability to quench fluorescence. Under optimized conditions for ZEN detection, the fluorescent aptasensor based on N-CDs/AuNPs showed a wide linear range from 0.25 to 200 ng/mL, with a low detection limit of 0.0875 ng/mL. The aptasensor demonstrated high sensitivity, specificity, and spiked recovery in quantitative ZEN detection. The method proved user-friendly with a short detection time, highlighting its suitability for rapid detection of ZEN in food samples.

## Figures and Tables

**Table 1:** Comparison of the fabricated assay with other methods for ZEN detection.

Method	Description	Linear Range (ng/mL)	LOD (ng/mL)	Refs.
Colorimetric	AuNPs	2.5~100	0.98	[35]
Colorimetric	AuNPs	10~250	10	[36]
Electrochemical	rGNR−rGO	0.25~500	0.09	[37]
Electrochemical	rGNRs/AuNPs	1~500	0.34	[38]
Fluorescence	MnO_2_	1.5~10	0.68	[39]
Fluorescence	CdTe QDs/Fe_3_O_4_	5~120	2.9	[40]
Fluorescence	AuNPs/N−CDs	0.25~200	0.0875	This work

**Table 2:** Determination of zearalenone in real samples (n = 3).

Sample	Spiked (ng/mL)	This Work			HPLC			The Correlations (R^2^)
		Detected (ng/mL)	Recovery (%)	CV (%)	Detected (ng/mL)	Recovery (%)	CV (%)	
Corn flour	50	51.76	103.52	3.47	53.30	106.60	1.85	0.9951
	100	90.92	90.67	6.69	96.14	96.14	1.53	
	200	169.50	84.75	5.18	194.02	97.01	2.07	
Corn oil	50	54.28	108.56	3.44	52.28	104.56	0.55	0.9804
	100	87.64	87.64	2.13	110.54	110.54	2.11	
	200	184.15	92.08	1.24	203.54	101.77	2.53	

## Data Availability

Data are contained within the article.

## References

[1] Liu Y., Yamdeu J.H.G., Gong Y.Y., Orfila C. (2020). A review of postharvest approaches to reduce fungal and mycotoxin contamination of foods. Compr. Rev. Food Sci. Food Saf..

[2] De Rycke E., Foubert A., Dubruel P., Bol O.I., De Saeger S., Beloglazova N. (2021). Recent advances in electrochemical monitoring of zearalenone in diverse matrices. Food Chem..

[3] Peters J., Ash E., Gerssen A., Van Dam R., Franssen M.C.R., Nielen M.W.F. (2021). Controlled Production of Zearalenone-Glucopyranoside Standards with Cunninghamella Strains Using Sulphate-Depleted Media. Toxins.

[4] Murtaza B., Jin B.W., Wang L.L., Li X.Y., Saleemi M.K., Majeed S., Khatoon A., Li G., Xu Y.P. (2023). Mitigation of zearalenone in vitro using probiotic strains. LWT-Food Sci. Technol..

[5] Kumar M., Singh G., Kaur N., Singh N. (2022). Organic Cation Receptor for Colorimetric Lateral Flow Device: Detection of Zearalenone in Food Samples. ACS Appl. Mater. Interfaces.

[6] Vidal-Acuña M.R., de Pipaón M.R.P., Torres-Sánchez M.J., Aznar J. (2018). Identification of clinical isolates of Aspergillus, including cryptic species, by matrix assisted laser desorption ionization time-of-flight mass spectrometry (MALDI-TOF MS). Med. Mycol..

[7] Kou Q.M., Wu P., Sun Q., Li C.X., Zhang L., Shi H.X., Wu J., Wang Y.R., Yan X.L., Le T. (2021). Selection and truncation of aptamers for ultrasensitive detection of sulfamethazine using a fluorescent biosensor based on graphene oxide. Anal. Bioanal. Chem..

[8] Kalra P., Dhiman A., Cho W.C., Bruno J.G., Sharma T.K. (2018). Simple Methods and Rational Design for Enhancing Aptamer Sensitivity and Specificity. Front. Mol. Biosci..

[9] Canoura J., Yu H.X., Alkhamis O., Roncancio D., Farhana R., Xiao Y. (2021). Accelerating Post-SELEX Aptamer Engineering Using Exonuclease Digestion. J. Am. Chem. Soc..

[10] Fan Y.T., Li J.X., Amin K., Yu H.S., Yang H.H., Guo Z.J., Liu J.S. (2023). Advances in aptamers, and application of mycotoxins detection: A review. Food Res. Int..

[11] Wang Y., Liu X.L., Wu L.J., Ding L.H., Effah C.Y., Wu Y.J., Xiong Y.M., He L.L. (2022). Construction and bioapplications of aptamer-based dual recognition strategy. Biosens. Bioelectron..

[12] Xu Y., Zhou C.Q., Li D.M., Guo C.X., Li Z.Y., Xing X.H., Li S.X., Guan T., Liu L., He Y.H. (2022). A stabilized weak measurement sensor for aptamer detection. Sens. Actuators B-Chem..

[13] Esmaelpourfarkhani M., Abnous K., Taghdisi S.M., Chamsaz M. (2020). A novel turn-off fluorescent aptasensor for ampicillin detection based on perylenetetracarboxylic acid diimide and gold nanoparticles. Biosens. Bioelectron..

[14] Cho H.H., Jung D.H., Heo J.H., Lee C.Y., Jeong S.Y., Lee J.H. (2023). Gold Nanoparticles as Exquisite Colorimetric Transducers for Water Pollutant Detection. ACS Appl. Mater. Interfaces.

[15] Hou S.L., Ma J.J., Cheng Y.Q., Wang Z.F., Yan Y.X. (2023). Overview-gold nanoparticles-based sensitive nanosensors in mycotoxins detection. Crit. Rev. Food Sci. Nutr..

[16] Wang M.J., Da Y., Tian Y. (2023). Fluorescent proteins and genetically encoded biosensors. Chem. Soc. Rev..

[17] Park J., Yang K.A., Choi Y., Choe J.K. (2022). Novel ssDNA aptamer-based fluorescence sensor for perfluorooctanoic acid detection in water. Environ. Int..

[18] Li J.M., Li S., Li Z.J., Zhou Y.T., Jin P., Zhang F.Y., Sun Q., Le T., Jirimutu (2023). Chromium hydroxide nanoparticles-based fluorescent aptameric sensing for sensitive patulin detection: The significance of nanocrystal and morphology modulation. Talanta.

[19] Sun Q., Li Z.J., Liu N.X., Zhou Y.T., Zhang F.Y., Li S., Jin P., Xiang R., Le T. (2023). Development of a novel fluorescent aptasensor based on the interaction between hexagonal β-Co(OH)_2_ nanoplates and nitrogen-doped carbon dots for ultrasensitive detection of patulin. Anal. Chim. Acta.

[20] Zhang X., Tan X.Y., Hu Y.P. (2021). Blue/yellow emissive carbon dots coupled with curcumin: A hybrid sensor toward fluorescence turn-on detection of fluoride ion. J. Hazard. Mater..

[21] Liu H.X., Zhong X., Pan Q., Zhang Y., Deng W.T., Zou G.Q., Hou H.S., Ji X.B. (2024). A review of carbon dots in synthesis strategy. Coord. Chem. Rev..

[22] Yin N., Yuan S., Zhang M., Wang J.Y., Li Y., Peng Y., Bai J.L., Ning B.A., Liang J., Gao Z.X. (2020). An aptamer-based fluorometric zearalenone assay using a lighting-up silver nanocluster probe and catalyzed by a hairpin assembly. Microchim. Acta.

[23] Qaddoori M.H., Al-Shmgani H.S. (2023). Galangin-Loaded Gold Nanoparticles: Molecular Mechanisms of Antiangiogenesis Properties in Breast Cancer. Int. J. Breast Cancer.

[24] Li J.M., Zhou Y.T., Li Z.J., Wang T., Sun Q., Le T., Jirimutu (2023). A novel fluorescent sensing platform based on nitrogen-doped carbon quantum dots for rapid and sensitive detection of aflatoxin B_1_ in corn flour. LWT-Food Sci. Technol..

[25] Tang X.D., Yu H.M., Bui B., Wang L.Y., Xing C., Wang S.Y., Chen M.L., Hu Z.Z., Chen W. (2021). Nitrogen-doped fluorescence carbon dots as multi-mechanism detection for iodide and curcumin in biological and food samples. Bioact. Mater..

[26] Yan Y., Liu J.H., Li R.S., Li Y.F., Huang C.Z., Zhen S.J. (2019). Carbon dots synthesized at room temperature for detection of tetracycline hydrochloride. Anal. Chim. Acta.

[27] Chen W.F., Shen J.L., Wang Z., Liu X., Xu Y.Y., Zhao H.Y., Astruc D. (2021). Turning waste into wealth: Facile and green synthesis of carbon nanodots from pollutants and applications to bioimaging. Chem. Sci..

[28] Ni P., Li Q.Y., Xu C.F., Lai H.Q., Bai Y., Chen T.F. (2019). Optical properties of nitrogen and sulfur co-doped carbon dots and their applicability as fluorescent probes for living cell imaging. Appl. Surf. Sci..

[29] Luo L.J., Li L.B., Xu X.X., Liu D., Li J.Y., Wang K., You T.Y. (2017). Determination of pentachlorophenol by anodic electrochemiluminescence of Ru(bpy)32^+^ based on nitrogen-doped graphene quantum dots as co-reactant. RSC Adv..

[30] Zhang W.J., Liu S.G., Han L., Luo H.Q., Li N.B. (2019). A ratiometric fluorescent and colorimetric dual-signal sensing platform based on N-doped carbon dots for selective and sensitive detection of copper (II) and pyrophosphate ion. Sens. Actuators B-Chem..

[31] John G.S.M., Vuttaradhi V.K., Takeuchi S., Pitani R.S., Venkatraman G., Rayala S.K. (2020). Facile synthesis and nanoscale features of a nanostructured nordihydroguaiaretic acid analog for therapeutic applications. J. Nanobiotechnol..

[32] Lerga T.M., Skouridou V., Bermudo M.C., Bashammakh A.S., El-Shahawi M.S., Alyoubi A.O., O’Sullivan C.K. (2020). Gold nanoparticle aptamer assay for the determination of histamine in foodstuffs. Microchim. Acta.

[33] Kabb C., Carmean R.N., Sumerlin B. (2016). Probing the surface-localized hyperthermia of gold nanoparticles in a microwave field using polymeric thermometers. Chem. Sci..

[34] Wang J.L., Wu Y.G., Zhou P., Yang W.P., Tao H., Qiu S.Y., Feng C.W. (2018). A novel fluorescent aptasensor for ultrasensitive and selective detection of acetamiprid pesticide based on the inner filter effect between gold nanoparticles and carbon dots. Analyst.

[35] Liu M., Zhang J.X., Liu S.S., Li B.X. (2022). A label-free visual aptasensor for zearalenone detection based on target-responsive aptamer-cross-linked hydrogel and color change of gold nanoparticles. Food Chem..

[36] Sun S.M., Zhao R., Feng S.M., Xie Y.L. (2018). Colorimetric zearalenone assay based on the use of an aptamer and of gold nanoparticles with peroxidase-like activity. Microchim. Acta.

[37] Zhou B.B., Xie H., Li X.Y., Zhu Y.B., Huang L.J., Zhong M., Chen L. (2024). Construction of a self-reporting molecularly-imprinted electrochemical sensor based on CuHCF modified by rGNR-rGO for the detection of zearalenone. Food Chem..

[38] Zhou B.B., Xie H., Zhou S.S., Sheng X.X., Chen L., Zhong M. (2023). Construction of AuNPs/reduced graphene nanoribbons co-modified molecularly imprinted electrochemical sensor for the detection of zearalenone. Food Chem..

[39] Qin G.X., Wei Y.W., Zhou Q.F., Wang H.J., Wei Y.N., Lao S.B., Luo L.H., Mo R.F., Chen Y.X., Yang Y.X. (2022). A sensitive MnO_2_ nanosheet sensing platform based on a fluorescence aptamer sensor for the detection of zearalenone. Anal. Methods.

[40] Liao X.L., Liu Y.M., Qiu L.Y., Cao L., Yang X.X., Hu X.G. (2023). A quantum dot aptamer fluorescent sensor based on magnetic graphene oxide for the detection of zearalenone. Anal. Methods.

